# The Functional Potential of Microbial Communities in Hydraulic Fracturing Source Water and Produced Water from Natural Gas Extraction Characterized by Metagenomic Sequencing

**DOI:** 10.1371/journal.pone.0107682

**Published:** 2014-10-22

**Authors:** Arvind Murali Mohan, Kyle J. Bibby, Daniel Lipus, Richard W. Hammack, Kelvin B. Gregory

**Affiliations:** 1 National Energy Technology Laboratory, Pittsburgh, Pennsylvania, United States of America; 2 Department of Civil and Environmental Engineering, Carnegie Mellon University, Pittsburgh, Pennsylvania, United States of America; 3 Department of Civil and Environmental Engineering, University of Pittsburgh, Pittsburgh, Pennsylvania, United States of America; 4 Department of Computational and Systems Biology, University of Pittsburgh Medical School, Pittsburgh, Pennsylvania, United States of America; Agriculture and Agri-Food Canada, Canada

## Abstract

Microbial activity in produced water from hydraulic fracturing operations can lead to undesired environmental impacts and increase gas production costs. However, the metabolic profile of these microbial communities is not well understood. Here, for the first time, we present results from a shotgun metagenome of microbial communities in both hydraulic fracturing source water and wastewater produced by hydraulic fracturing. Taxonomic analyses showed an increase in anaerobic/facultative anaerobic classes related to *Clostridia*, *Gammaproteobacteria*, *Bacteroidia* and *Epsilonproteobacteria* in produced water as compared to predominantly aerobic *Alphaproteobacteria* in the fracturing source water. The metabolic profile revealed a relative increase in genes responsible for carbohydrate metabolism, respiration, sporulation and dormancy, iron acquisition and metabolism, stress response and sulfur metabolism in the produced water samples. These results suggest that microbial communities in produced water have an increased genetic ability to handle stress, which has significant implications for produced water management, such as disinfection.

## Introduction

High-volume hydraulic fracturing operations for natural gas development from deep shale produce millions of gallons of wastewater over the lifetime of the well [1], [2], [3], commonly termed as ‘produced water’. This produced water contains elevated concentrations of salts, metals, hydrocarbons and radioactive elements [3], [4], [5], [6], [7]. Microbial communities in produced water can utilize hydrocarbons as sources of carbon and energy [8] and transform redox labile salts and metals. This can give rise to significant water management challenges [9] and increased production costs [10], [11]. For instance, sulfidogenic and acid producing bacteria can cause corrosion of metal infrastructure, souring of natural gas, and reduced formation permeability [10], [11], [12], [13].

Deleterious microbial activity is commonly controlled with biocides at significant cost to the driller. However, despite biocide use, microbial activity is prevalent in produced water. Previous studies have shown that biocide effectiveness may be limited by high salt concentrations, organic compounds, and long residence times in the subsurface [14], [15], [16]. Other studies have shown that microbial communities in produced water are distinct from those in the injected fracturing fluid, and correlate well with changes in geochemical and environmental conditions [5], [15], [17]. This implies that the common practice of recycling produced water for subsequent hydraulic fracturing may introduce adapted populations into the formation [5].

Over the past decade molecular ecology surveys based on the 16S rRNA gene have increased our knowledge about the taxonomic composition of microbial communities in reservoir environments [5], [15], [17], [18], [19], [20], [21], [22]. However, these studies offer limited insights on the metabolic capabilities of the microbial community, as they rely on taxonomic inference based on 16S rRNA gene similarity to previously isolated microorganisms. As an example of the limitations of using previously isolated microorganisms to infer metabolic capability, the ‘core genome’ of the well-studied *Escherichia coli* is typically less than 50% of the genes in the genome, and <30% of the *E. coli* pan-genome [23]. On the other hand, shotgun metagenomic surveys enable access to complete genetic information within microbial genomes from uncultured, mixed consortia [24], [25], [26]. These surveys have provided significant insights on the functional potential of microorganisms in diverse environments such as marine samples [25], corals [27], activated sludge [28], permafrost [29], hydrocarbon and sandstone reservoirs [30], [31], and swine gut [32]. Despite the importance of microbial activity in produced water brines from hydraulic fracturing operations, the functional potential of associated microbial communities has not yet been studied. In this study, the metagenome of fracturing source water and produced water at two different time points from a Marcellus Shale natural gas well in Westmoreland County, PA was generated using Illumina MiSeq technology. The microbial ecology from 16S rRNA surveys and chemical composition of these samples has been described in a previous publication [5]. Sequences from each sample were assembled into contiguous sequences (contigs) and analyzed for taxonomic affiliations and functional potential of the microbial communities.

## Materials and Methods

### Sampling

Samples of hydraulic fracturing source water, and produced water on days 1 and 9 were collected from a horizontally drilled Marcellus Shale natural gas well in Westmoreland County, Pennsylvania, U.S.A in October 2011. The source water used for fracturing was a mix of fresh reservoir water (∼80%) and produced water (∼20%) from previous fracturing operations. Fracturing additives amended to the source water included proppant (silica sand), scale inhibitor (ammonium chloride), biocide (mixture of tributyl tetradecyl phosphonium chloride, methanol and proprietary chemicals), hydrochloric acid, gel (paraffinic solvent), breaker (sodium persulfate) and friction reducer (hydrotreated petroleum distillate). Details regarding the sampling procedure and chemical additives used in the fracturing process are described elsewhere [5]. The aqueous geochemical characteristics of these samples were described previously [5] (Table S1).

### DNA extraction, library preparation and Illumina sequencing

Unfiltered water samples were centrifuged at 6,000 *g* for 30 min in an Avanti J-E centrifuge (Beckman Coulter, Brea, CA) to pellet cells. DNA was extracted from 0.25 g of cell pellet using MO BIO power soil DNA isolation kit (MO BIO, Carlsbad, CA) according to the manufacturer's instructions. DNA was prepared using Nextera XT DNA sample preparation kit (Illumina, San Diego, CA) according to manifacturer's instructions at Genewiz (South Plainfield, NJ). DNA for sequencing was quantified using qPCR prior to clustering, and sequenced using the Illumina MiSeq (Illumina, San Diego, CA) with a 2×250 PE configuration at Genewiz, NJ. Sequencing demultiplexing was performed on the Illumina MiSeq instrument using sample-specific barcodes.

### Bioinformatic analyses

The raw unpaired sequences were checked for sequencing tags and adapters using the predict function implemented within the TagCleaner program [33]. No sequencing tags or adapters were identified. Sequences were then subjected to quality control using the FastX toolkit within the Galaxy platform [34] with a minimum length 100 and minimum quality score 20. The velvet assembler [35] was used to assemble sequences that passed quality control into contiguous sequences. The assembly parameters were empirically optimized for the dataset prior to assembly (Table S2); the dataset was processed using a kmer length of 77. Generated contigs >500 bp in length were uploaded to the MG-RAST server [36] with associated metadata files for taxonomic affiliations and functional annotations. Sequence similarity searches in MG-RAST was performed using the BLAT tool [37]. The metagenomes from fracturing source water, day 1 produced water, and day 9 produced water are available in the MG-RAST server [36] under accession nos. 4525703.3, 4525704.3 and 4525705.3, respectively. Taxonmic assignments of selected funcional categories from MG- RAST were excecuted in MGTAXA [38], [39], on the Galaxy bioinformatics workbench [40], [34], using default parameters and taxonomy as defined by the NCBI taxonomic tree. Data is for contig abundance and does not reflect read mapping.

As an additional assembly-independent analysis, sequence data was mapped against reference genomes downloaded from NCBI (Table S3) with CLC Genomics Workbench (Version 6.5.1, CLC Bio, Aarhus, Denmark) [41] using default parameters and no masking. Reference genomes were selected based upon taxonomic observations in MG-RAST annotation and a previous microbial ecology investigation [5]. Prior to mapping, sequencing data was trimmed to a minimum length of 100 bp and minimum quality score of 20. Furthremore, sequences for the sulfite reductase subunits A and B (dsrA/dsrB) (Table S4a) and the suflur metabolism gene adenylyl sulfate reductase subunit A (apsA) (Table S4b) were downloaded from NCBI and mapped against the trimmed sequencing data using CLC Genomics Workbench (Version 6.5.1, CLC Bio, Arhus, Denmark).

## Results and Discussion

A total of 10 002, 17 055 and 16 661 contigs from the fracturing source water, produced water day 1 and day 9 samples, respectively, were uploaded to MG-RAST for downstream analyses. All uploaded contigs passed MG-RAST quality control and de-replication filters. The metagenomics sequence statistics are summarized in Table 1.

**Table 1:** Metagenomic sequence statistics of fracturing source water (SW), produced water day 1 (PW day 1) and produced water day 9 (PW day 9).

	SW	Pw day 1	PW day 9
Total base pair (bp) count	7,939,565 bp	18,254,354 bp	15,253,129 bp
No. of Contigs	10,002	17,055	16,661
Mean length of Contigs	793±809 bp	1,070±1,195 bp	915±651 bp
% GC content in Contigs	59±8%	55±13%	43±9%
% Contigs containing predicted proteins with known functions	83%	93.1%	80.8%
% Contigs containing predicted proteins with unknown functions	16.6%	6.6%	18.9%
% Contigs containing rRNA genes	0.4%	0.3%	0.3%
Identified protein features	9,919	20,687	16,982
Identified functional categories	8,041	16,948	13, 570

### Taxonomic composition

Taxonomic affiliations were assigned to contigs with predicted proteins and rRNA genes based on comparison with the M5NR database. Alpha diversity (predicted phylotypes) for the fracturing source water, produced water day 1 and day 9 samples were 90, 79 and 88, respectively (Figure S1). Rarefaction curves for each of the samples were asymptotic suggesting that the majority of taxonomic diversity was recovered from the samples (Figure S1). Alpha diversity values and rarefaction curves were obtained using the MG-RAST tool.

**Figure 1:**
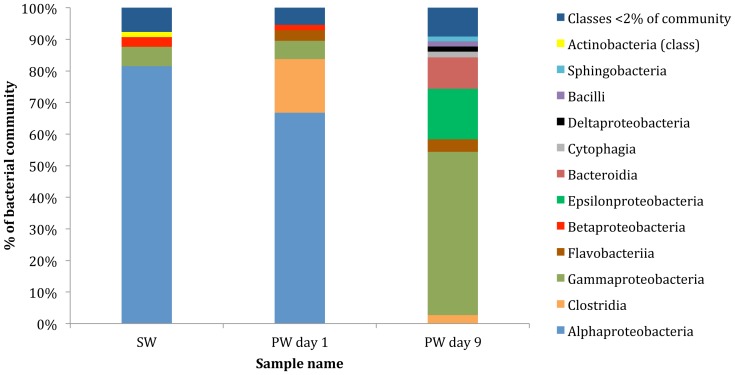
Class level affiliations assigned to contigs with predicted proteins and rRNA genes in source water (SW), produced water day 1 (PW day 1) and produced water day 9 (PW day 9). Total community includes *Bacteria*, *Archaea*, *Viruses* and *Eukaryota*.

**Figure 2:**
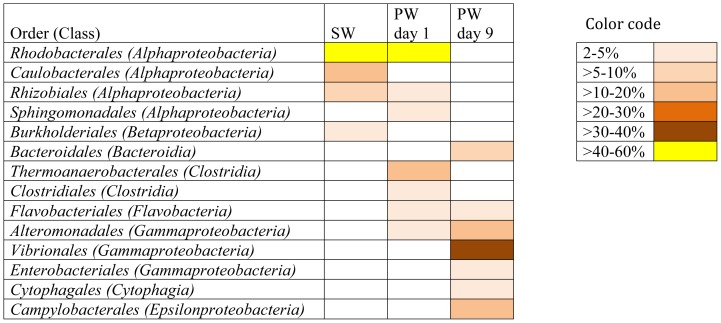
Order level affiliations assigned to contigs with predicted proteins and rRNA genes in source water (SW), produced water day 1 (PW day 1) and produced water day 9 (PW day 9). Total community includes *Bacteria*, *Archaea*, *Viruses* and *Eukaryota*. Only orders representing >2% of the total community are shown in the figure.

*Bacteria* constituted the dominant domain (97–99% of the total community) in all samples. However, a shift in bacterial community composition was detected between the samples at the class and order levels (Figure 1, 2). Contigs affiliated to the class *Alphaproteobacteria* constituted the majority of the community in the fracturing source water (81%) and produced water day 1 (67%) samples (Figure 1). Within *Alphaproteobacteria*, the dominant order detected was *Rhodobacterales* (68–88% of the *Alphaproteobacteria*; 55–59% of the total community) in both the source water and produced water day 1 samples (Figure 2). The relative abundance of *Alphaproteobacteria* decreased to <2% of the community in the produced water day 9 sample. Previous qPCR analysis of these samples suggests that that the total bacterial population remained constant at 10^6^–10^7^ copies of 16S RNA gene/ml [5].

An increase in the number of contigs associated with the class *Clostridia* was observed in the produced water day 1 sample (17%) as compared to the fracturing source water (1%). However, the relative abundance of *Clostridia* decreased to 3% in the produced water day 9 sample. The majority of the *Clostridia* in the produced water day 1 sample were affiliated to the order *Thermoanaerobacterales* (94% of *Clostridia*; 16% of the total community) (Figure 2). *Gammaproteobacteria* sequences constituted a minor fraction (6%) of the total community in the fracturing source water and produced water day 1 samples but increased in relative abundance to constitute the dominant class (52%) in the produced water day 9 sample. Within the *Gammaproteobacteria* of the produced water day 9 sample, dominant orders included *Vibrionales* (67% of *Gammaproteobacteria*) and *Alteromonadales* (23% of *Gammaproteobacteria*) (Figure 2). The day 9 samples also showed an increase in relative abundance of *Epsilonproteobacteria* (16%) and *Bacteroidia* (10%) classes as compared to the other samples (<2% of the total community). The major bacterial phyla, classes and orders identified in this study were consistent with previous 16S rRNA gene based clone library and pyrosequencing surveys of these samples (Figure S2) [5]. These results indicate a shift towards facultative anaerobic/anaerobic and halophilic communities in the produced water samples as compared to a predominantly aerobic community in the fracturing source water. At the class level, in each of the samples less that 3% of the total sequences did not affiliate to any taxonomic group.

A minor fraction of the total community was represented by contigs affiliated to *Archaea* (0.1–0.4%), *Viruses* (0.3–1%) and *Eukaryota* (0.4–1.4%) domains. These domains were not analyzed for in the previous 16S rRNA gene survey of these samples [5], and were not considered in more detailed functional classification of the metagenomes.

### Mapping results

**Figure 3:**
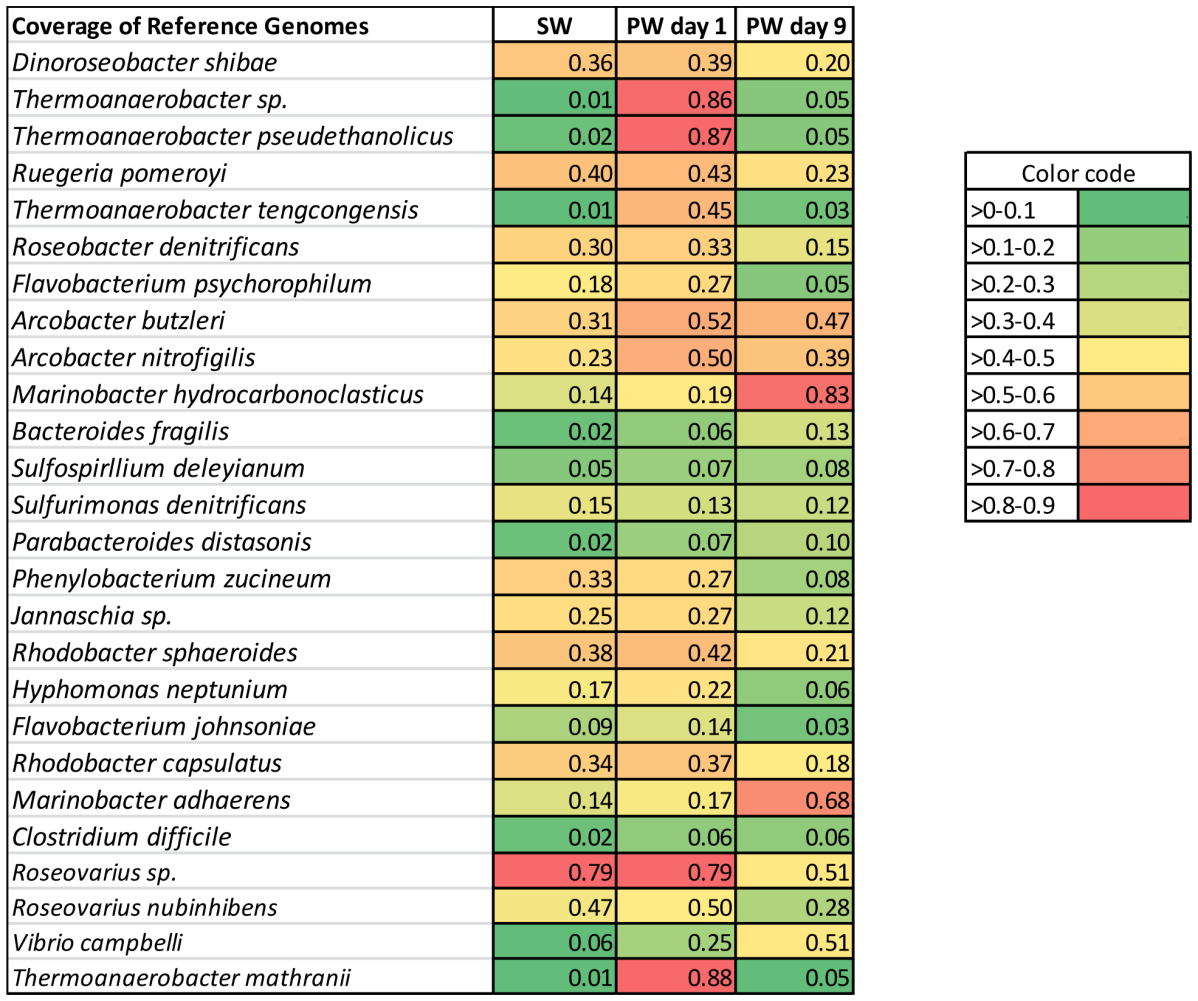
Fraction of genome coverage for source water (SW), produced water day 1 (PW day 1) and produced water day 9 (PW day 9) samples. Reads were mapped against reference genomes using CLC Genomic workbench version 6.5.1 using default parameters. Shown are fractions of reads mapped against each reference genome included in the analysis for all three samples.

**Figure 4:**
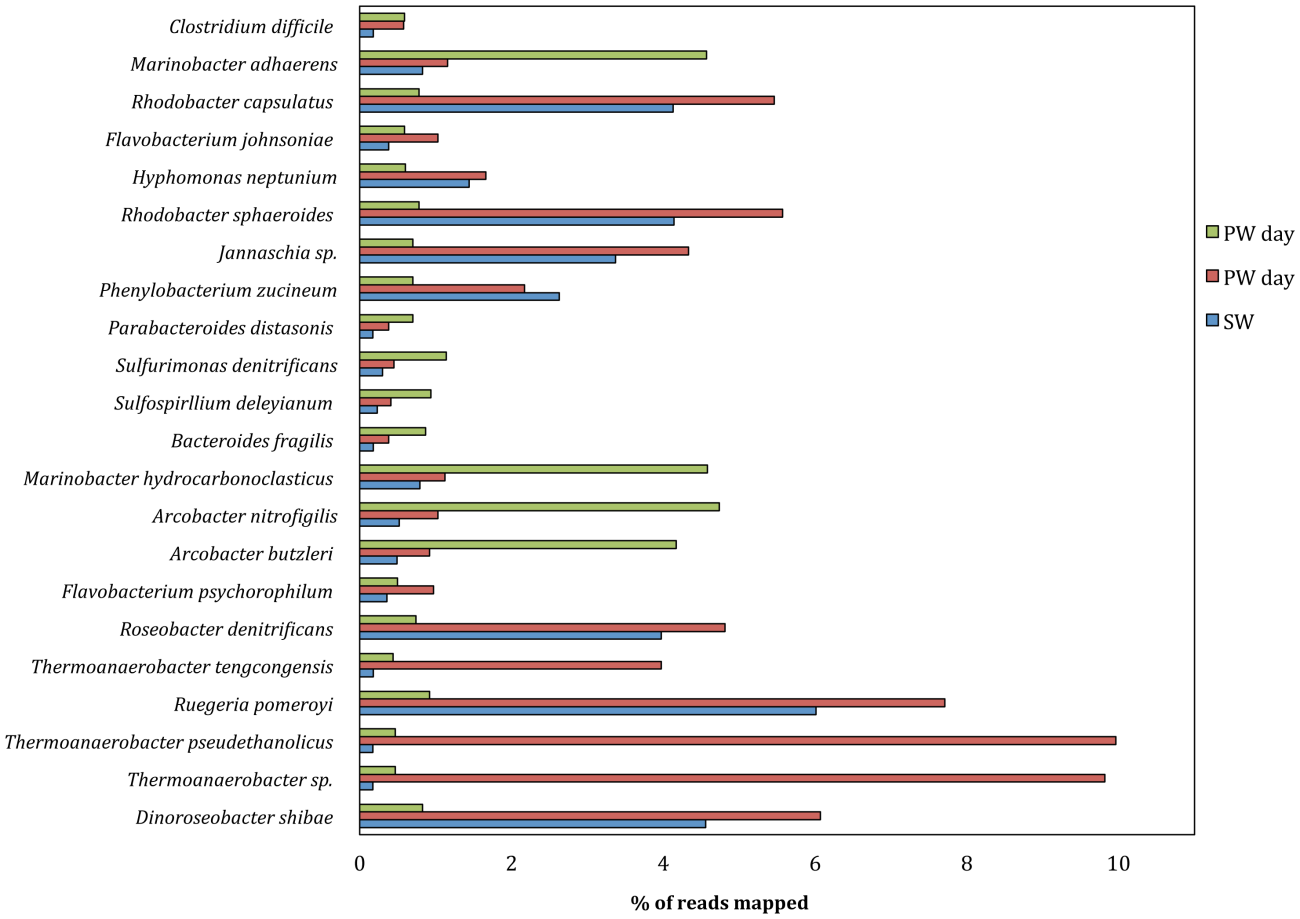
Read distribution for source water (SW), produced water day 1 (PW day 1) and produced water day 9 (PW day 9) samples. Reads were mapped against reference genomes using CLC Genomic workbench version 6.5.1 using default parameters. Shown are percentages of reads mapped against each reference genome included in the analysis for all three samples.

Metagenomic reads were mapped against a diverse set of reference genomes to confirm MG-RAST taxonomic results and only reference genomes with good mapping results are discussed in this section. Reference genome mapping results confirmed taxonomic MG-RAST contig analysis. The best mapping results for source water were obtained when sequences were mapped against reference genomes of *Alphaproteabacteria*, specifically of the order *Rhodobacterales* (Figures 3, 4). Similarly, produced water day 1 sample mapping results suggest that it was dominated by bacteria of the orders *Rhodobacterales* and *Thermoanaerobacterales* (Figures 3, 4). A distinct shift in bacterial community was observed between produced water day 1 samples and produced water day 9 samples based on mapping results. Best mapping results for produced water day 9 samples were obtained for reference genomes in the order *Campylobacterales* and *Alteromondales* further supporting the MG-RAST results (Figures 3, 4). Produced water samples demonstrated a distinctive signature with reads mapping best to few select reference genomes, while source water sample reads were distributed more evenly throughout all included reference genomes. For four reference genomes (*Thermoanaerobacter sp.* X514, *Thermoanaerobacter pseudethanolicus*, *Thermoanaerobacter mathranii* in produced water day 1 samples and *Marinobacter hydrocarbonoclasticus* DSM 7299 *i*n produced water day 9 sample) more than 80% coverage was achieved suggesting that these species could play important roles in the microbial community of the representative sample (Figure 3). Highest observed reference genome coverage for source water sample sequences were 79% for *Roseovarius* sp. 217, 40% for *Ruegeria pomeroyi* and 38% for *Rhodobacter sphaeroides* (Figure 3). For produced water day 1 samples, about 10% of all trimmed sequencing reads mapped against the three *Thermanaerobacter* genomes included in the analysis and 8–13% of reads mapped successfully against *Roseovarius sp.* 217 and *Roseovarius nubinhibens* genomes (Figure 4). 7.7% of produced water day 1 reads mapped against the *Ruegeria pomeroyi* genome (Figure 4). 4–6% of reads for produced water day 9 samples mapped against two different *Marinobacter* and *Arcobacter* reference genomes and one *Vibrio* reference genome (Figure 4). Almost 16% of all reads from source water samples mapped against *Roseovarius sp.* 217 and approximately 4–6% of reads for source water sample mapped against each *Dinoroseobacter shibae*, *Ruegeria pomeroyi*, *Rhodobacter sphaeroides* and *Rhodobacter capsulatus* genomes (Figure 4). All mapping results are summarized in Table S3. The high number of reads form source water and produced water day 1 samples mapping against *Roseovarius* species is in agreement with previous 16S rRNA gene sequencing [5], implying the *Roseovarius* species might be of importance in these waters. *Roseovarius sp.* was previously identified in natural gas brines from the Marcellus shale and its potential implications are discussed elsewhere [9].

The goal of this analysis was to provide an independent confirmation of MG-RAST results. Mapping results depend on the reference genomes selected and these reference genomes might not be the same isolates found in the environment. While reference genomes for uncultured microorganisms from oil/gas environments are limited, the positive results achieved by this mapping analysis confirm the initial taxonomic assessment.

### Sulfur metabolism gene mapping results

Very few reads in all three samples were successfully mapped against the sulfur metabolism genes dsrA and dsrB. 7 reads of produced water day 1 sample and 55 reads of produced water day 9 sample were successfully mapped against the dsrA/dsrB gene of *Desulfovibrio desulfuricans* with a coverage of 28% and 78% respectively (Table S4a). In addition 10 reads of produced water day 9 sample were successfully mapped against the dsrA/dsrB gene of *Desulfotignum balticum* with a coverage of 19% (Table S4a). For aspA genes, the produced water day 9 sample showed best results with 16, 11, 9 and 6 reads successfully mapped against aspA genes of *Desulfovirbio alaskensis*, *Desulfococcus mulitvorans*, *Desulfotignum balcticum* and *Desulfobacterium autotorphicum* with a coverage of 94%, 46%, 33% and 31% respectively (Table S4b). Very few source water and produced water day 1 reads were mapped successfully against the aspA genes included in the analysis (Table S4b). These results suggest that sulfur metabolism could play a more important role in produced water day 9 sample due the higher abundance of genes associated with sulfur metabolism. Organisms that can metabolize sulfur compounds to sulfide are of interest in oil and gas environments because of their potential role in infrastructure corrosion, gas souring, worker safety as well as environmental health concerns.

### Functional classification of metagenomes

**Figure 5:**
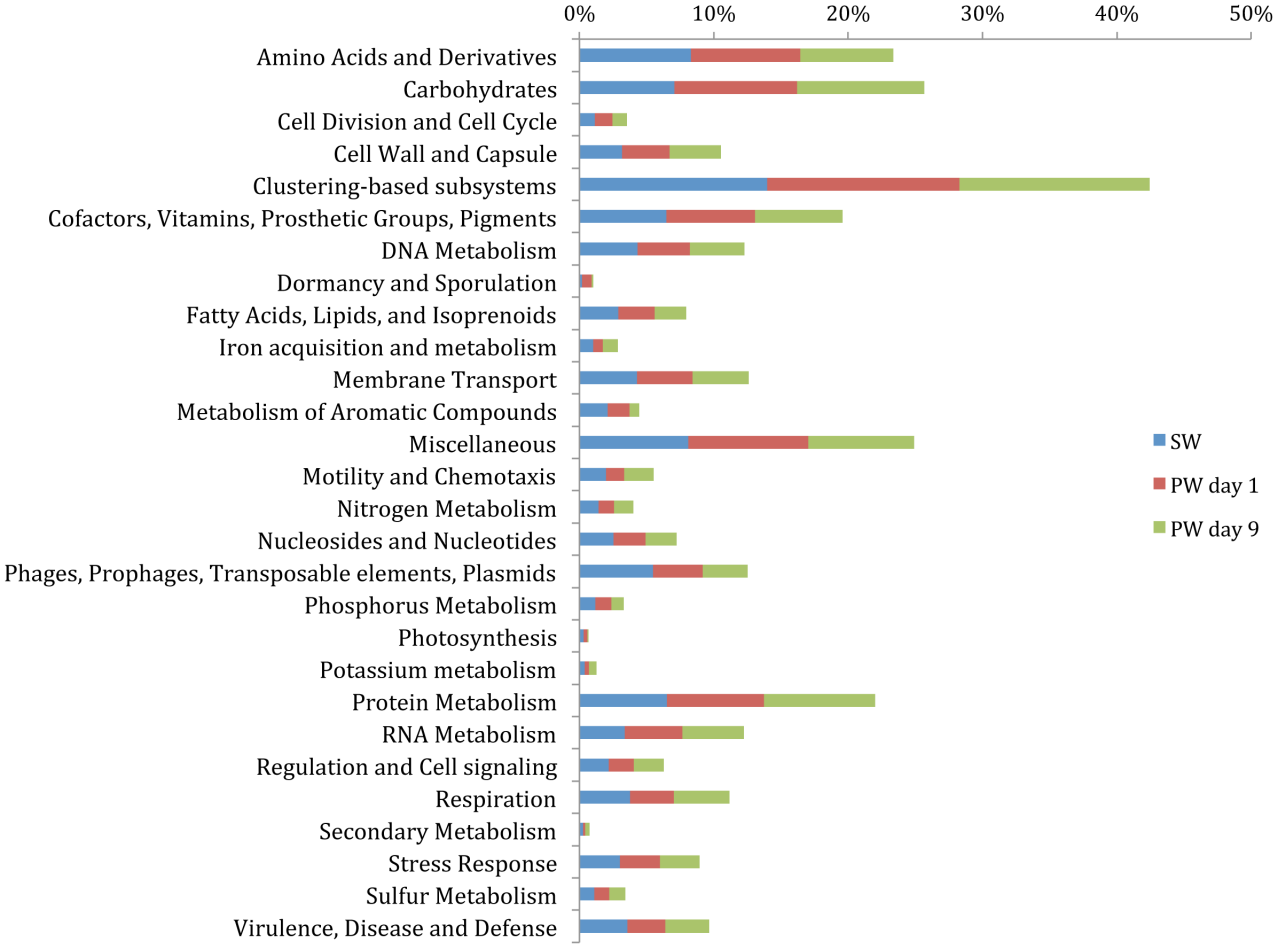
Actual abundance of contigs belonging to Level 1 functional categories in source water (SW), produced water day 1 (PW day 1) and produced water day 9 (PW day 9). Functional annotations were assigned based on the Subsystems database.

**Figure 6:**
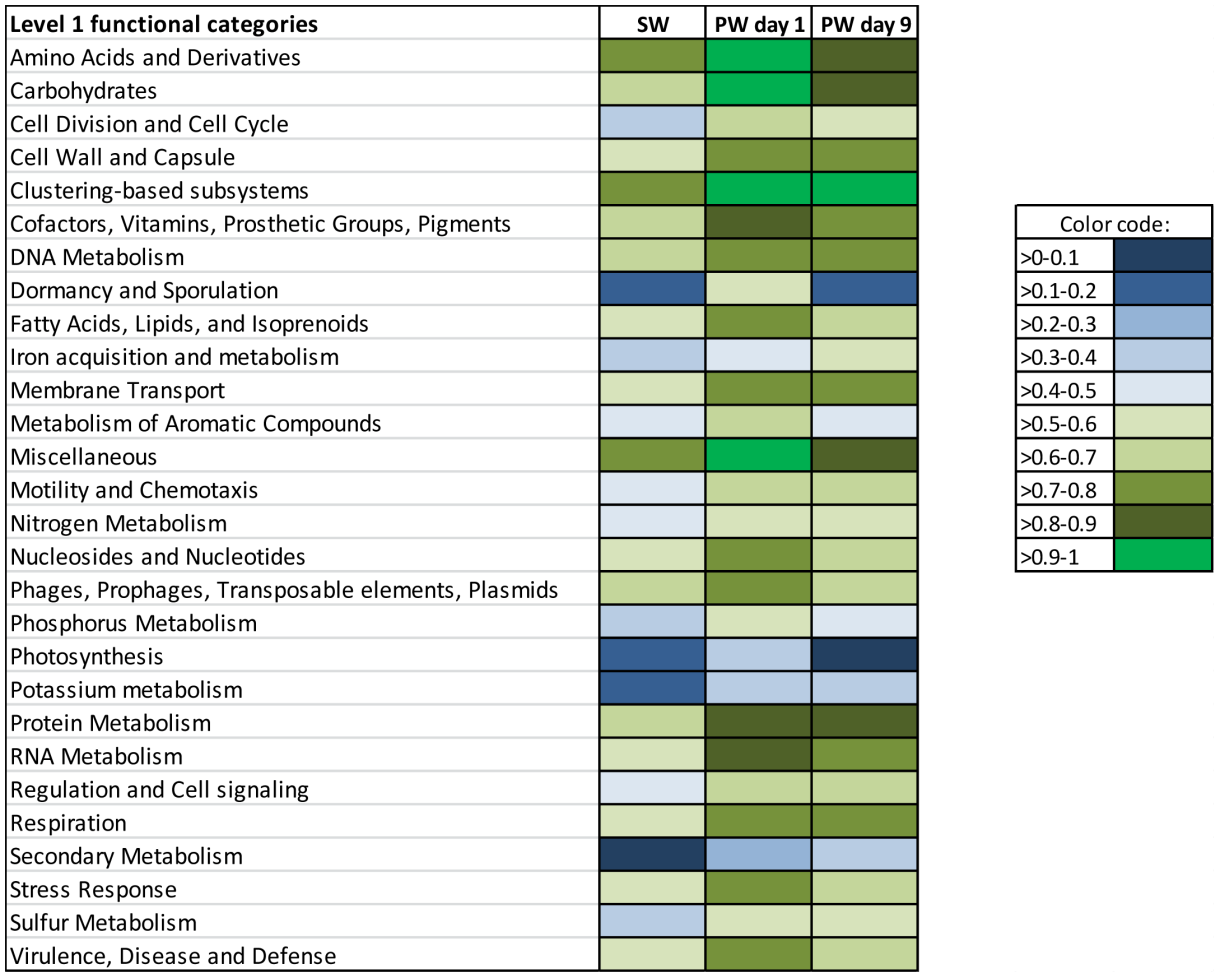
Normalized abundance (values of 0–1) of contigs belonging to Level 1 functional categories in source water (SW), produced water day 1 (PW day 1) and produced water day 9 (PW day 9). Functional annotations were assigned based on the Subsystems database.

**Figure 7:**
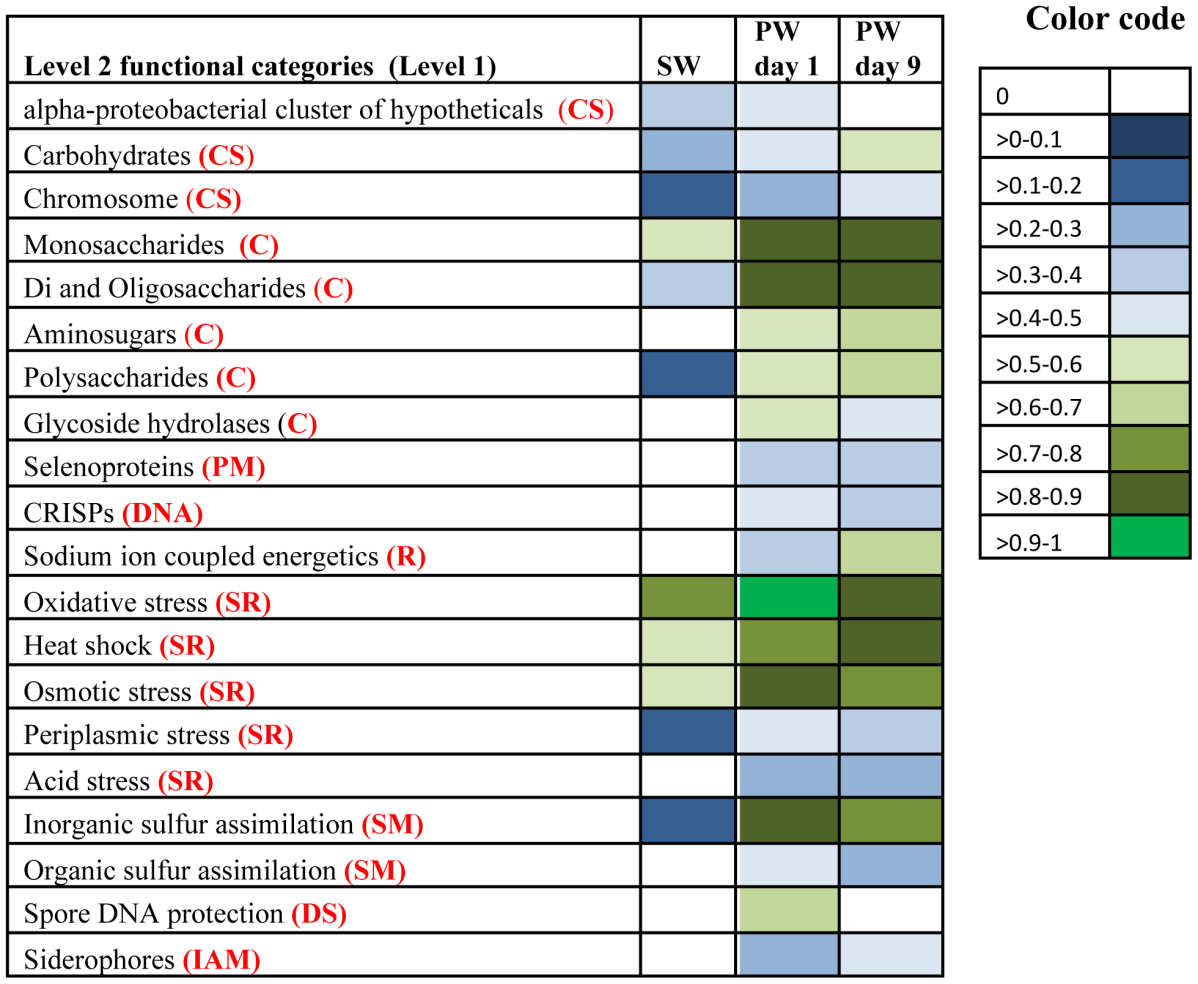
Normalized abundance (values of 0–1) of contigs belonging to selected Level 2 functional categories within associated Level 1 categories in source water (SW), produced water day 1 (PW day 1) and produced water day 9 (PW day 9). Functional annotations were assigned based on the Subsystems database. The affiliations of Level 2 categories to Level 1 categories are coded as follows CS- Clustering based subsystems; C- Carbohydrates; PM- Protein metabolism; DNA- DNA metabolism; R- Respiration; SR- Stress response; SM- Sulfur metabolism; DS- Dormancy and sporulation; IAM- Iron acquisition and metabolism.

The SEED subsystems database [42], was used to predict the metabolic potential of fracturing source water and produced water samples. Level 1 indicates the broadest set of functional categories to which sequences are assigned, and Level 2 refers to more specific functional assignments within Level 1 categories. The abundance of contigs designated to Level 1 functional categories is illustrated in Figure 5. The metabolic potential (based on Level 1 and Level 2 functional categories) between the samples was compared in a normalized manner (Figure 6, 7) to account for differences in community structure, size of the library, gene content between samples and to effectively compare low abundance functional categories [43]. Read normalization was performed within the MG-RAST analysis pipeline, in accordance with standards for metagenomic analysis.

The five most abundant Level 1 functional categories in all three samples were found to be clustering-based subsystems (e.g. genes where functional coupling is evident but function is unknown;∼14%), carbohydrate metabolism (7–9%), amino acids and derivatives (7–8%), miscellaneous (eg: genes associated with iron sulfur cluster assembly and Niacine-Choline transport and metabolism; 8–9%), protein metabolism (6–8%), suggesting the dominant role of these functional categories in all samples (Figure 5). These functional categories were similarly identified as dominant in previous studies of soil [44], [45], marine samples [24],[46], activated sludge [24], freshwater [24] and hypersaline environments [24]. Normalization of gene abundance data shows a relative increase in each of the above functional categories in the produced water samples as compared to the fracturing source water (Figure 6) implying that core systems necessary for survival are enriched in the produced water community.

**Figure 8:**
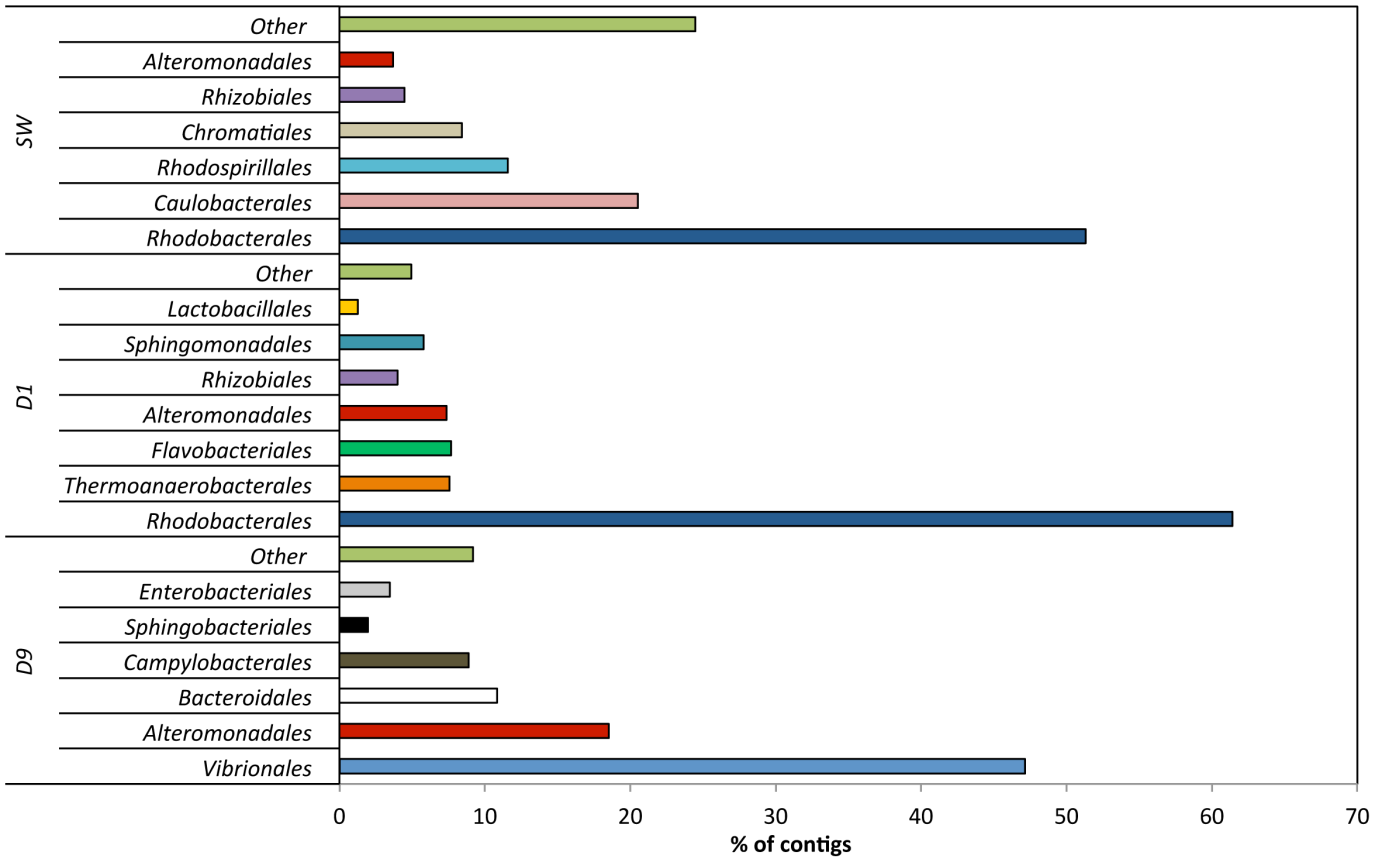
Taxonomic classification of oxidative stress contigs for each analyzed water sample as assigned by MGTAXA. SW- Source water; D1- Produced water day 1; D9- Produced water day 9. Only the top six bacterial orders to which most contigs were assigned to are shown in the figure. The less abundant bacterial orders are grouped as “other”.

While comparison of gene abundance affiliated with the dominant broad Level 1 categories suggests similar functional profiles across samples, analysis of more specific Level 2 functional categories shows sample specific differences in metabolic capabilities (Figure 7). Differences in metabolic potential indicate a selective pressure exerted in the subsurface for microbes with particular metabolic capabilities. For instance, within the Level 1 carbohydrate metabolism category, sequences related to Level 2 functional categories such as mono-, di-, oligo- and polysaccharides, and aminosugar metabolism were present in higher relative abundance in the produced water samples (Figure 7). This finding correlates well with the expected higher content of carbohydrates in produced water samples [5]. Carbohydrates and polysaccharide compounds added during hydraulic fracturing can serve as carbon and energy sources for microbial activity [8]. Within the Level 1 protein metabolism category, sequences affiliated with the Level 2 selenoprotein category were detected only in the produced water samples (Figure 7). One possible explanation is the role of selenoproteins in combating oxidative stress [47], which may arise from elevated concentrations of organic or inorganic dissolved constituents in produced water [48]. Results showed that *Rhodobacterales* were the dominant population involved in oxidative stress response in source water and produced water day 1 samples (Figure 8). However, *Alteromonadales* and *Vibrionales* were the dominant orders involved in oxidative stress response in produced water day 9 sample (Figure 8). Within the Level 1 clustering subsystem, genes affiliated with the Level 2 carbohydrate metabolism show a relative increase in the produced water samples as compared to fracturing source water (Figure 7). An increase in the relative abundance of genes related to carbohydrate metabolism in produced water compared to fracturing source water suggests the potential for utilization of hydrocarbons added either as fracturing fluid amendments or those derived from the shale formation and an overall shift to a more heterotrophic microbial community.

**Figure 9:**
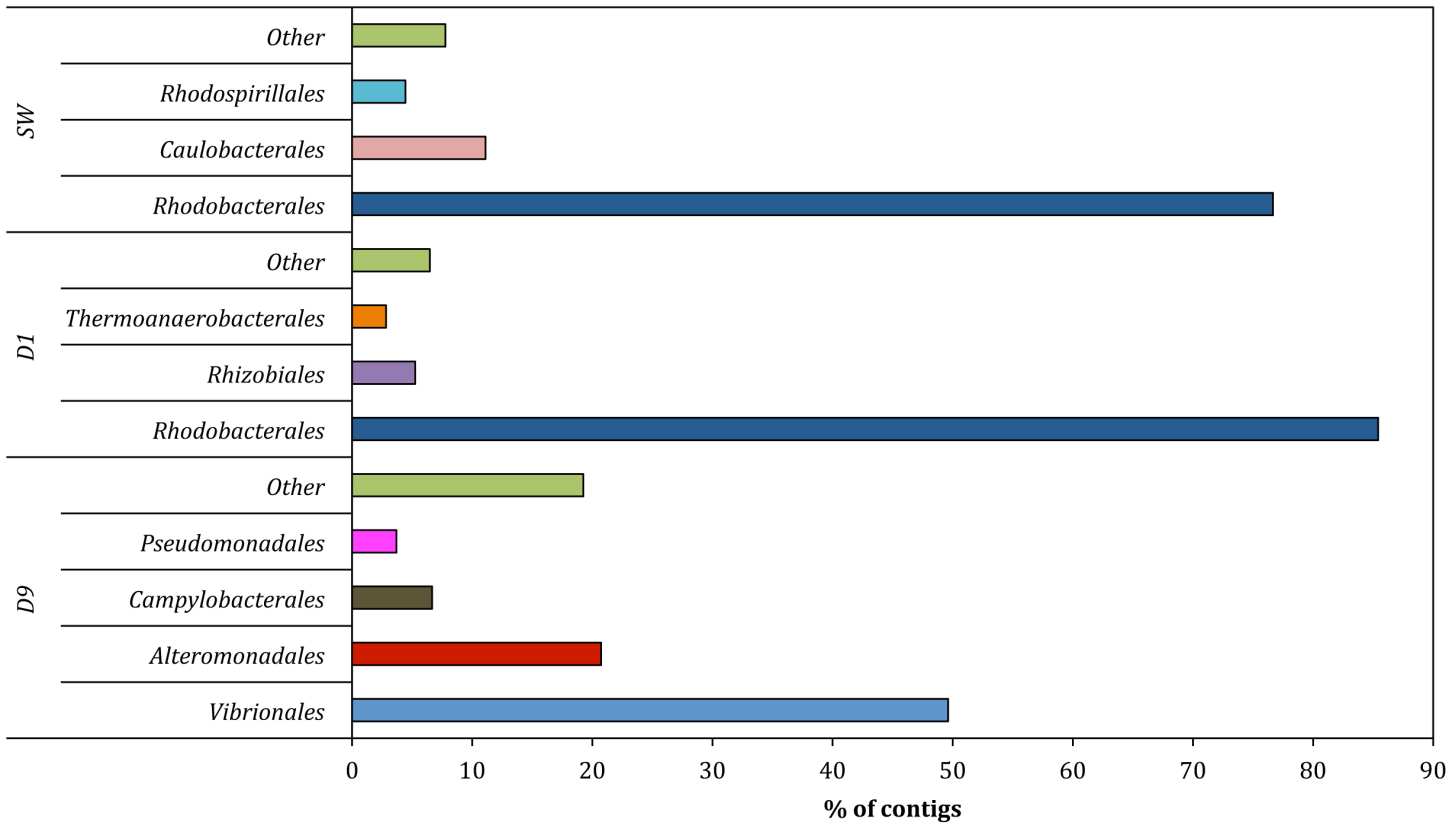
Taxonomic classification of osmotic stress contigs for each analyzed water sample as assigned by MGTAXA. SW- Source water; D1- Produced water day 1; D9- Produced water day 9. Only the top four bacterial orders to which most contigs were assigned to are shown in the figure. The less abundant bacterial orders are grouped as “other”.

**Figure 10:**
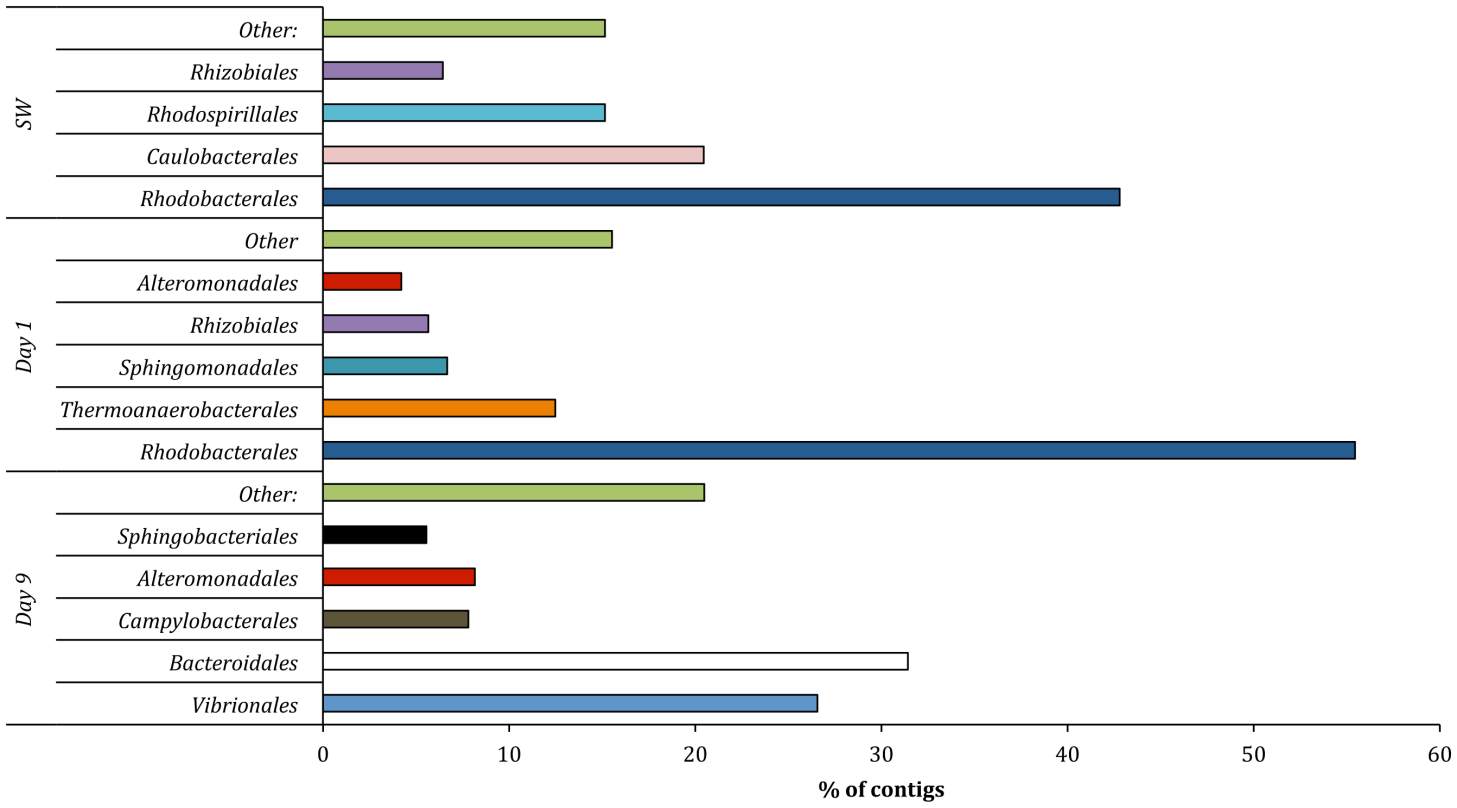
Taxonomic classification of sulfur metabolism contigs for each analyzed water sample as assigned by MGTAXA. SW- Source water; D1- Produced water day 1; D9- Produced water day 9. Only the top five bacterial orders to which most contigs were assigned to are shown in the figure. The less abundant bacterial orders are grouped as “other”.

Less abundant Level 1 functional categories showing an increase in normalized abundance in produced water samples (Figure 6) included genes affiliated with stress response (3%), respiration (3–4%), iron acquisition and metabolism (1%), sulfur metabolism (1%), and dormancy and sporulation (0.2–1%). Analysis of Level 2 functional categories within these Level 1 domains identified differences in metabolic potential between these samples (Figure 7). Within the Level 1 stress response domain, produced water samples showed a greater relative abundance of sequences affiliated with Level 2 categories such as acid stress, heat shock, periplasmic stress and osmotic stress (Figure 7). The increase in the relative abundance of these genes suggests a response to external stress experienced by the produced water microbial community. Results suggest that produced water day 1 population involved in osmotic stress response was dominated by the order *Rhodobacterales* and produced water day 9 population involved osmotic stress response was dominated by the orders *Vibrionales* and *Alteromonadales* (Figure 9). Subsurface stresses can include increased subsurface temperatures (>40°C) [49], addition of HCl and biocides to fracturing fluid, and higher concentrations of dissolved salts (Table S1) [5]. Within the Level 1 respiration category, sequences affiliated to the Level 2 category of sodium ion coupled energetics were undetected in fracturing source water (Na^+^ 2.9 g/L) but increased in relative abundance with time in produced water samples (Na^+^ concentrations in PW day 1 and day 9 were 13.9 and 43 g/L) (Figure 7). This suggests that the produced water microbial community could use sodium ion coupled energetics for their energy needs, consistent with previous observations in saline environments [50]. In the Level 1 domain of sulfur metabolism, the relative abundance of genes affiliated with Level 2 functional categories of inorganic and organic sulfur assimilation increased in produced water samples as compared to fracturing source water (Figure 7). Genes recovered from produced water day 1 show that populations involved in sulfur metabolism were dominated by the orders *Rhodobacterales* and *Thermoanaerobacterales* (Figure 10). However, sulfur metabolism in produced water day 9 samples was dominated by the orders *Vibrionales* and *Bacteroidales* (Figure 10). Within the Level 1 domain of iron metabolism, sequences affiliated with siderophores, undetected in the fracturing source water, increased with time in produced water samples (Figure 7). Siderophores are strong chelators of ferric iron secreted and are utilized by bacteria for iron metabolism [51]. Relative increase in siderophore affiliated genes correlates with an increase in total iron concentrations with time in produced water (4.2–81.6 mg/L) (Table S1). Within the Level 1 dormancy and sporulation category, high relative abundance of Level 2 spore DNA protection related sequences in produced water day 1 sample (Figure 7) suggests the potential for long term dormancy of cells through DNA protection [52]. BLAT analysis [37] showed that these genes were similar to those present in *Thermoanaerobacter*, a bacterial order that constituted 16% of the total community in this sample (Figure 2). An increase in the relative abundance of spore forming bacteria and genes affiliated with sporulation and dormancy is an important consideration in biocide application, and may provide an explanation for the previously observed limited efficacy of biocides [5].

#### Concluding Remarks

This study is the first shotgun metagenomic analysis of produced water from hydraulic fracturing for natural gas production and provides novel insights on taxonomic and functional potential of this pertinent yet unexplored environment. Taxonomic analysis showed that *Bacteria* constituted the dominant (>98%) domain in both fracturing source water and produced water samples. Results demonstrated the emergence of distinct bacterial classes and orders in the produced water samples and fracturing source water samples. These bacterial taxa were consistent with results from a previous 16S rRNA gene based survey of these samples [5]. The metabolic profile showed both a relative increase and functional changes in genes responsible for carbohydrate metabolism, respiration, sporulation and dormancy, iron acquisition and metabolism, stress response and sulfur metabolism in the produced water samples as compared to the fracturing source water sample. These results suggest that the microbial community is responsive to changes in hydrocarbon content, induced stresses such as increase in temperature, addition of biocides, and an increase in concentration of dissolved salts such as iron and sulfur. The detection of genes affiliated with sodium ion coupled energetics exclusively in the produced water samples suggests the use of sodium ion based energetics by microorganisms in these sodium rich environments. Understanding the evolving metabolic capabilities of microbial communities in produced water will help the industry and its regulators improve environmental and economic sustainability of oil and gas extraction through more informed water management decisions.

## Supporting Information

Figure S1 **Plot of refraction curves with associated Alpha diversity in fracturing source water (SW), produced water day 1 (PW day 1) and produced water day 9 (PW day 9).**
(TIF)

Figure S2 **Sequences affiliated to major bacterial phyla in source water, Produced water day 1 and Produced water day 9 using 16S rRNA gene pyrosequencing and metagenomics.**
(TIF)

Table S1 **Chemical composition of source water and produced water (PW) samples days 1, 9 and 187.**
(TIF)

Table S2 **Assembly optimization statistics.** Velvet 1.2.08 was used to optimize assembly of Source Water derived sequences.
(TIF)

Table S3 **Mapping results for source water, produced water day 1 and produced water day 9 sequencing data against selected bacteria species reference genomes.** Mapping analysis was performed using CLC Genomics Workbench version 6.5.1 with default parameters.
(TIF)

Table S4 **Mapping results, (A), for source water, produced water day 1 and produced water day 9 sequencing data against the genome sequences of the dsrA/dsrB gene of selected microbial organisms.** Mapping analysis was performed using CLC Genomics Workbench version 6.5.1 with default parameters. (B) Mapping results for source water, produced water day 1 and produced water day 9 sequencing data against the genome sequences of the apsA gene of selected microbial organisms. Mapping analysis was performed using CLC Genomics Workbench version 6.5.1 with default parameters.
(TIF)
