# Supplementary figures and images for: The Functional Potential of Microbial Communities in Hydraulic Fracturing Source Water and Produced Water from Natural Gas Extraction Characterized by Metagenomic Sequencing
Source: PLoS One. 2014 Oct 22;9(10):e107682. doi: 10.1371/journal.pone.0107682 (PMC4206270; doi:10.1371/journal.pone.0107682)

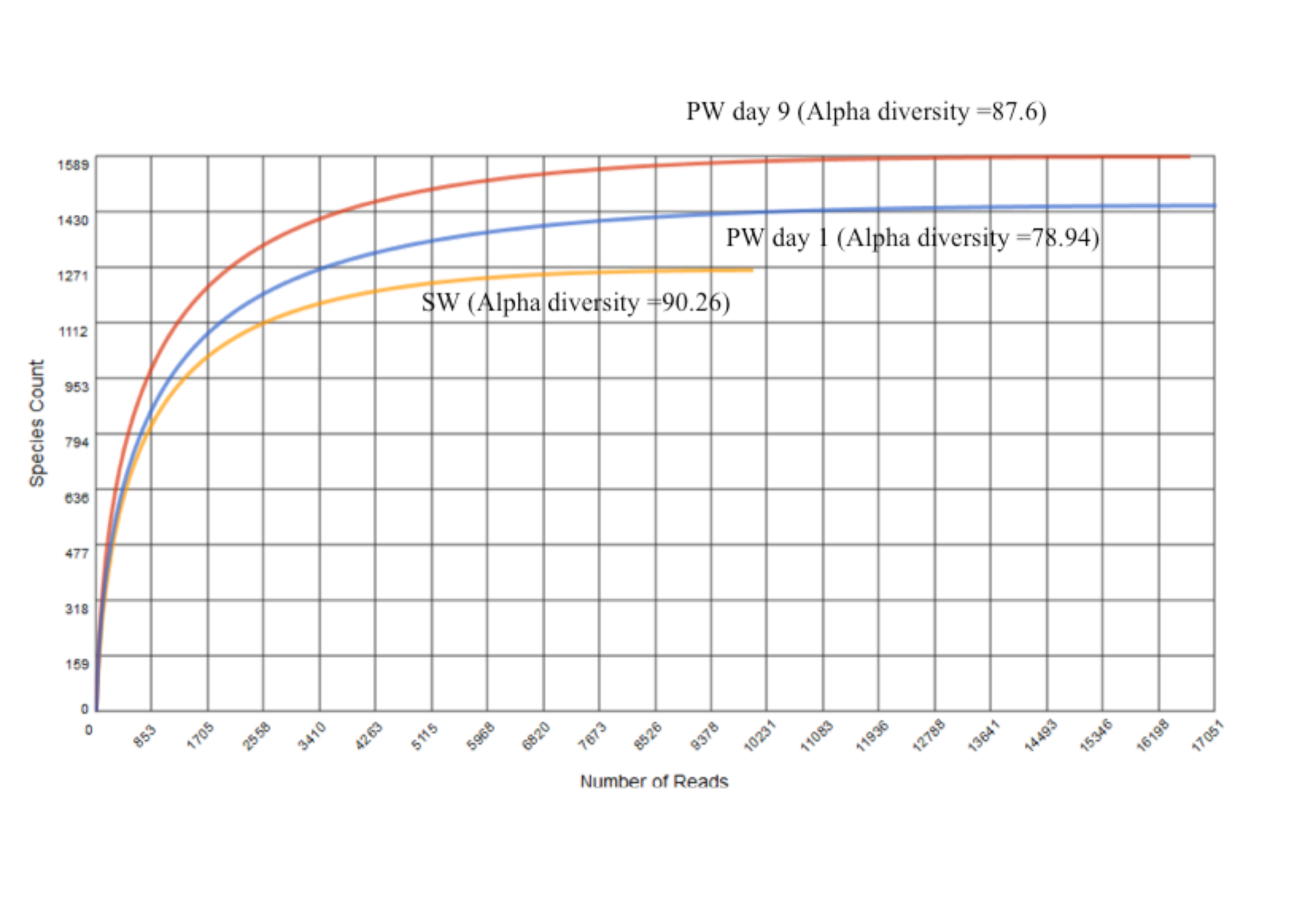

Supplement: Figure S1 — Plot of refraction curves with associated Alpha diversity in fracturing source water (SW), produced water day 1 (PW day 1) and produced water day 9 (PW day 9). (TIF) [file pone.0107682.s001.tif]

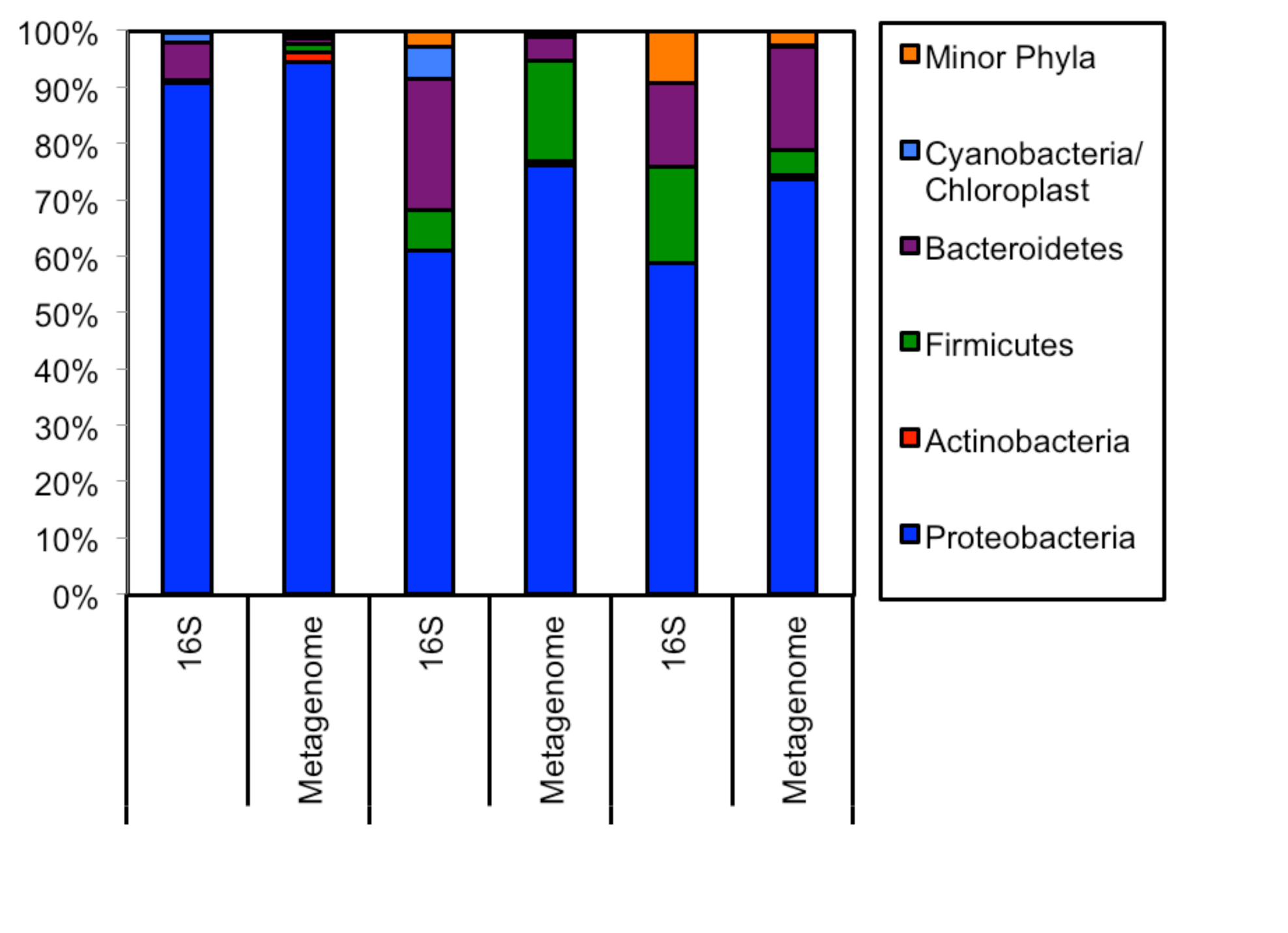

Supplement: Figure S2 — Sequences affiliated to major bacterial phyla in source water, Produced water day 1 and Produced water day 9 using 16S rRNA gene pyrosequencing and metagenomics. (TIF) [file pone.0107682.s002.tif]

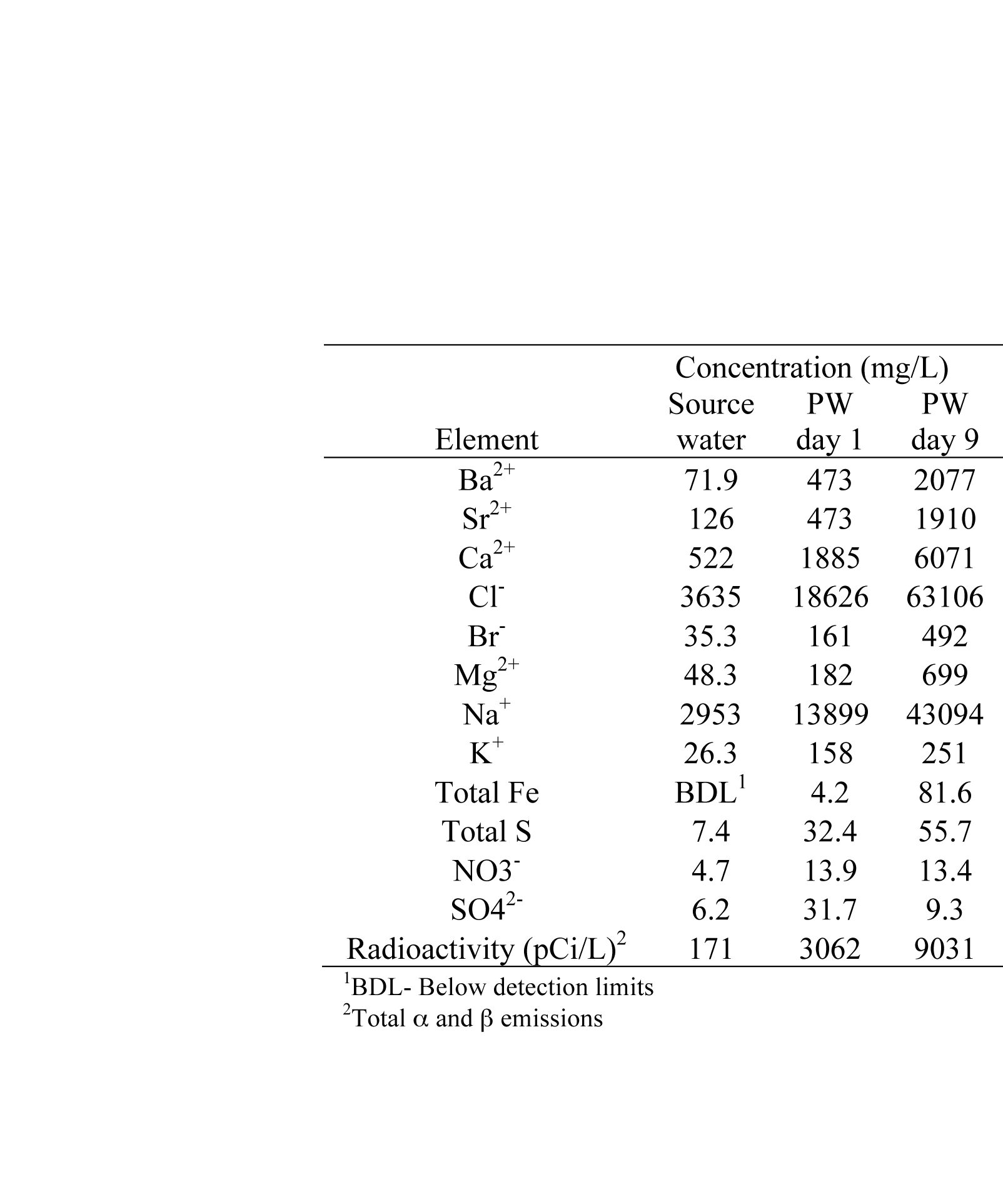

Supplement: Table S1 — Chemical composition of source water and produced water (PW) samples days 1, 9 and 187. (TIF) [file pone.0107682.s003.tif]

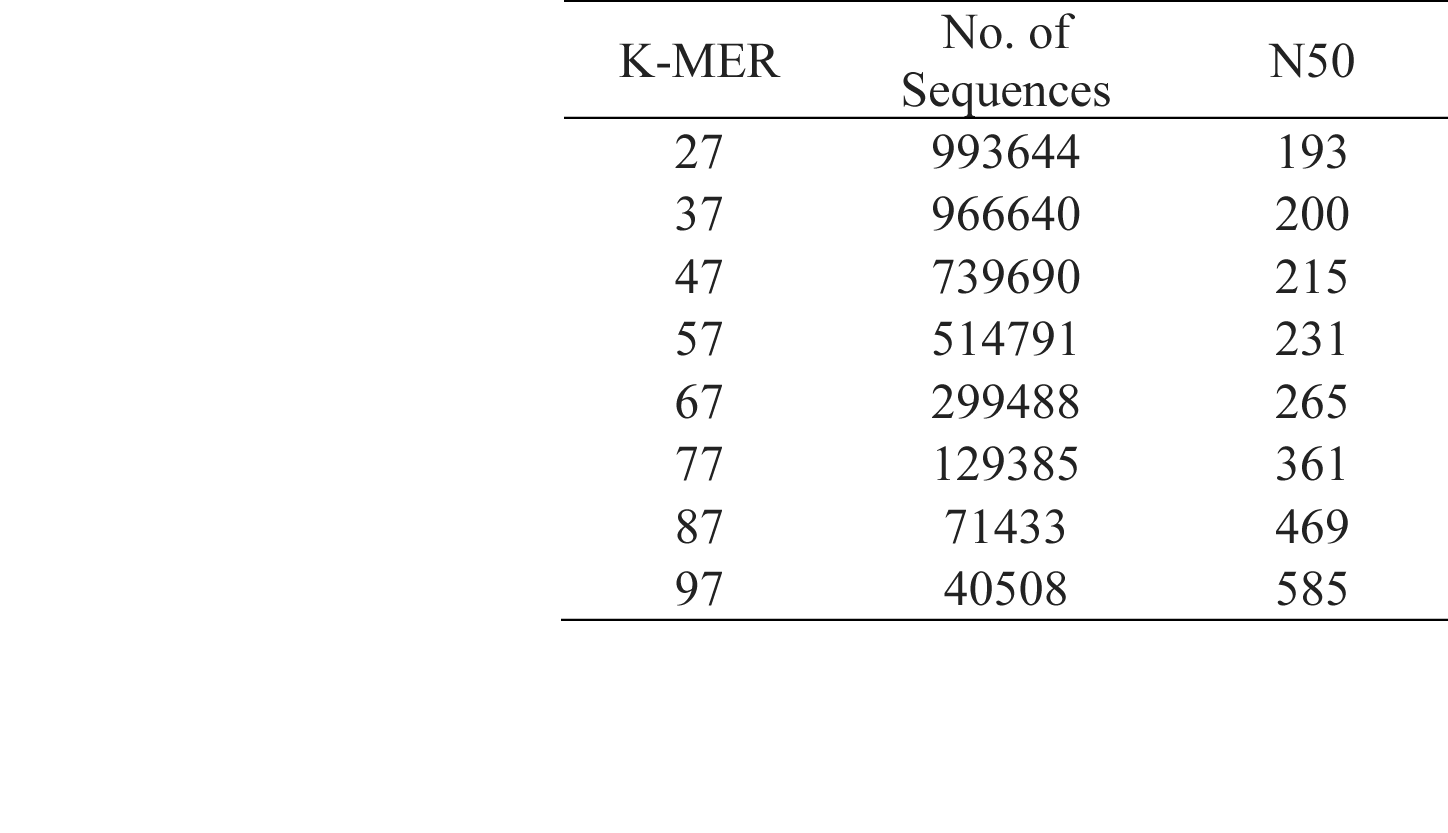

Supplement: Table S2 — Assembly optimization statistics. Velvet 1.2.08 was used to optimize assembly of Source Water derived sequences. (TIF) [file pone.0107682.s004.tif]

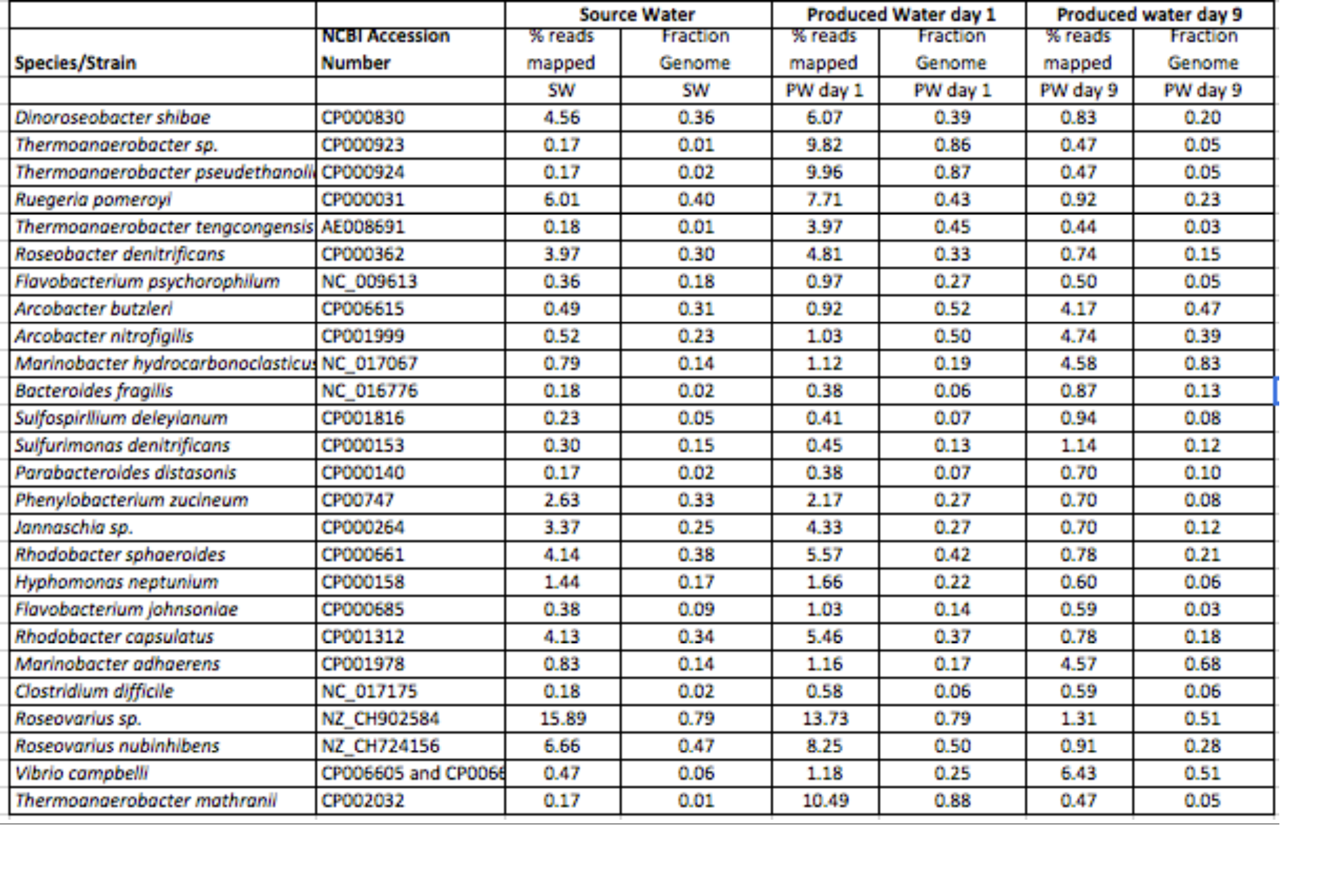

Supplement: Table S3 — Mapping results for source water, produced water day 1 and produced water day 9 sequencing data against selected bacteria species reference genomes. Mapping analysis was performed using CLC Genomics Workbench version 6.5.1 with default parameters. (TIF) [file pone.0107682.s005.tif]

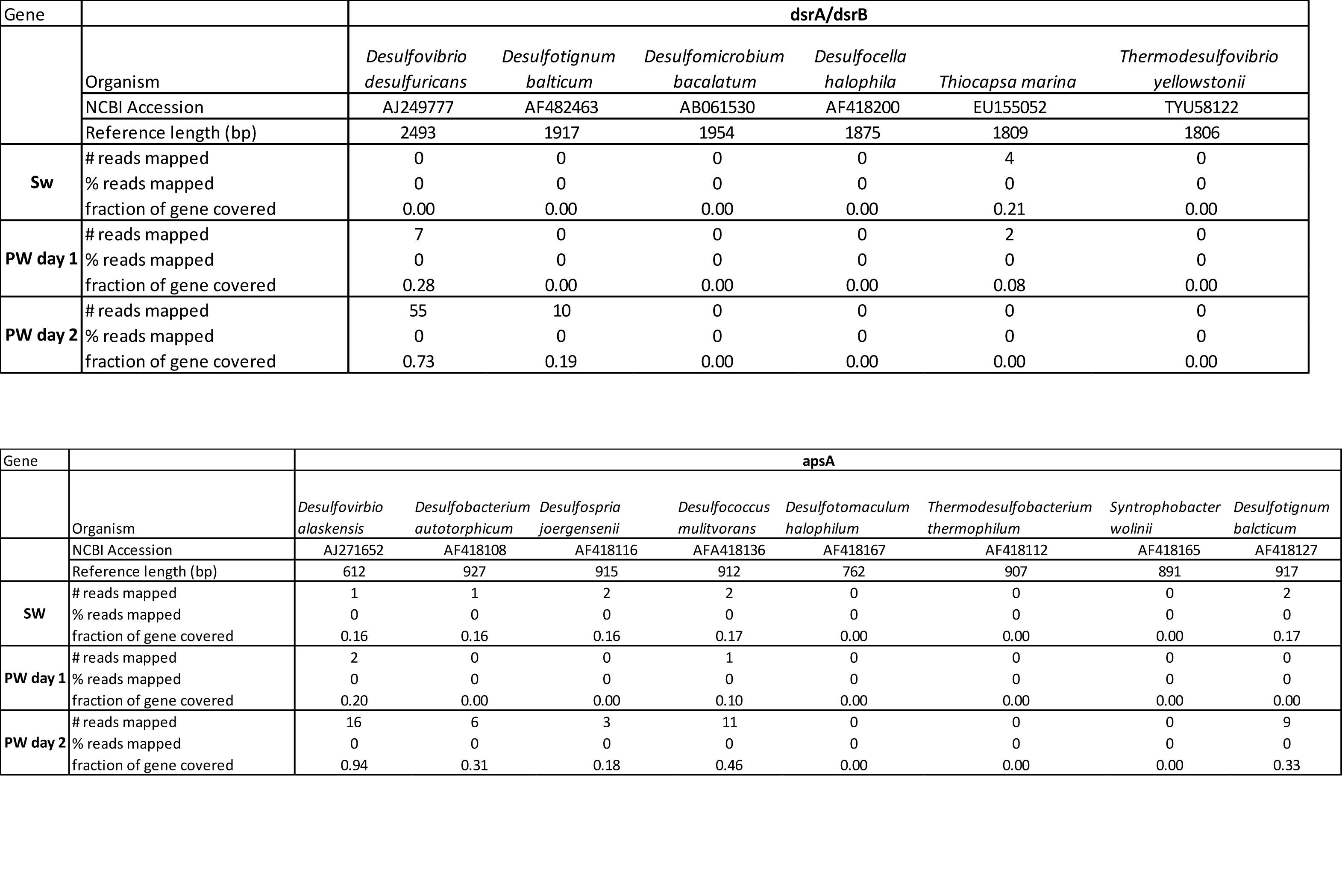

Supplement: Table S4 — Mapping results, (A), for source water, produced water day 1 and produced water day 9 sequencing data against the genome sequences of the dsrA/dsrB gene of selected microbial organisms. Mapping analysis was performed using CLC Genomics Workbench version 6.5.1 with default parameters. (B) Mapping results for source water, produced water day 1 and produced water day 9 sequencing data against the genome sequences of the apsA gene of selected microbial organisms. Mapping analysis was performed using CLC Genomics Workbench version 6.5.1 with default parameters. (TIF) [file pone.0107682.s006.tif]
